# Efficient Anomaly Detection with Generative Adversarial Network for Breast Ultrasound Imaging

**DOI:** 10.3390/diagnostics10070456

**Published:** 2020-07-04

**Authors:** Tomoyuki Fujioka, Kazunori Kubota, Mio Mori, Yuka Kikuchi, Leona Katsuta, Mizuki Kimura, Emi Yamaga, Mio Adachi, Goshi Oda, Tsuyoshi Nakagawa, Yoshio Kitazume, Ukihide Tateishi

**Affiliations:** 1 Department of Diagnostic Radiology, Tokyo Medical and Dental University, 1-5-45 Yushima, Bunkyo-ku, Tokyo 113-8510, Japan; kubotard@dokkyomed.ac.jp (K.K.); m_mori_116@yahoo.co.jp (M.M.); 11.ruby.89@gmail.com (Y.K.); leonah@jcom.home.ne.jp (L.K.); 150421ms@tmd.ac.jp (M.K.); ymgdrnm@tmd.ac.jp (E.Y.); ktzmmrad@tmd.ac.jp (Y.K.); ttisdrnm@tmd.ac.jp (U.T.); 2 Department of Radiology, Dokkyo Medical University, 880 Kitakobayashi, Mibu, Shimotsugagun, Tochigi 321-0293, Japan; 3 Department of Surgery, Breast Surgery, Tokyo Medical and Dental University, 1-5-45 Yushima, Bunkyo-ku, Tokyo 113-8510, Japan; mioadachi1016@gmail.com (M.A.); oda.srg2@tmd.ac.jp (G.O.); nakagawa.srg2@tmd.ac.jp (T.N.)

**Keywords:** breast imaging, ultrasound, deep learning, anomaly detection, generative adversarial network

## Abstract

We aimed to use generative adversarial network (GAN)-based anomaly detection to diagnose images of normal tissue, benign masses, or malignant masses on breast ultrasound. We retrospectively collected 531 normal breast ultrasound images from 69 patients. Data augmentation was performed and 6372 (531 × 12) images were available for training. Efficient GAN-based anomaly detection was used to construct a computational model to detect anomalous lesions in images and calculate abnormalities as an anomaly score. Images of 51 normal tissues, 48 benign masses, and 72 malignant masses were analyzed for the test data. The sensitivity, specificity, and area under the receiver operating characteristic curve (AUC) of this anomaly detection model were calculated. Malignant masses had significantly higher anomaly scores than benign masses (*p* < 0.001), and benign masses had significantly higher scores than normal tissues (*p* < 0.001). Our anomaly detection model had high sensitivities, specificities, and AUC values for distinguishing normal tissues from benign and malignant masses, with even greater values for distinguishing normal tissues from malignant masses. GAN-based anomaly detection shows high performance for the detection and diagnosis of anomalous lesions in breast ultrasound images.

## 1. Introduction

Breast cancer is the most common cancer and the second leading cause of cancer death among women [1]. Ultrasound is a widely used modality for detecting and diagnosing breast cancer when other imaging modalities such as mammography and clinical examination find abnormalities. Ultrasound is considered a leading imaging modality because of its high availability, cost effectiveness, acceptable diagnostic performance, and noninvasive real-time capabilities [2,3,4].

The breast imaging reporting and data system lexicon [5] was developed by the American College of Radiology to standardize terms for the description and classification of breast lesions and was reported to show good diagnostic performance. However, the diagnosis of images relies on the experience of radiologists. Therefore, significant intra- and inter-individual variabilities may occur [6]. In addition, the fatigue and stress of the radiologist can lead to overlooked findings and misdiagnoses [7].

Recently, a deep learning (DL) method demonstrated better performance than standard computer vision algorithms in medical imaging for pattern recognition, segmentation, object detection, and image synthesis. DL was recently applied to breast imaging modalities such as mammography, ultrasound, and magnetic resonance imaging (MRI) [8,9,10,11,12,13,14,15]. Sophisticated DL models have the potential to help radiologists diagnose patients efficiently.

However, the establishment of DL models in clinical applications is impeded by several barriers. Because most DL models rely on supervised learning, they can only be applied to data that resemble a training set. In addition, models based on supervised training have limitations when applied to the diagnosis of rare diseases because it is difficult to collect sufficient data on any given rare disease to train the model sufficiently [16].

Detection of anomalies is one of the most important aspects in various fields, such as manufacturing, medical imaging, and cyber security [17,18]. For anomaly detection, a learning model can be developed using only normal data, and it does not require complicated, supervised data. Therefore, such a model can be used for patients with unknown heterogeneous distribution. In recent years, an excellent anomaly detection model based on generative adversarial network (GAN) has been described [19,20,21]. GAN is a special type of neural network computational model in which two networks are trained simultaneously: one focuses on image generation and the other on discrimination [22]. However, to the best of our knowledge, there has been no report of clinical application of a GAN-based anomaly detection model to analyze breast ultrasound images. We therefore aimed to use this model to diagnose images of normal tissue, benign masses, and malignant masses on breast ultrasound.

## 2. Materials and Methods

### 2.1. Patients

Our institution’s medical ethics committee (Tokyo Medical and Dental University Hospital Ethics Committee) approved this retrospective study (approval ID: M2019-232, approval date: 13 December 2019) and waived the requirement for obtaining informed consent from patients. The inclusion criteria for patient enrollment were as follows: (1) patients who underwent breast ultrasound at our hospital between March 2014 and October 2019 and (2) patients who were diagnosed with a normal, benign, or malignant status by histopathology at a follow-up period of >1 year. The following patients were excluded: (1) those who were treated with breast surgery, hormonal therapy, chemotherapy, or radiation therapy and (2) those who were aged <20 years. After reviewing the clinical records and radiology report database, a research assistant (M.K.) randomly selected breast ultrasound images.

### 2.2. Breast Ultrasound Examinations

Ultrasound was performed by one of five radiologists with 4–21 years of experience in breast ultrasound. The equipment used included an Aplio XG scanner with a PLT-805AT 8.0-MHz linear probe (Toshiba Medical Systems, Tochigi, Japan), an Aplio 500 scanner with a PLT-805AT 8.0-MHz linear probe (Toshiba Medical Systems, Tochigi, Japan), or an EUB-7500 scanner with a EUP-L54MA 9.75-MHz linear probe (Hitachi Medical Systems, Tokyo, Japan). The radiologists acquired multiple static images of normal mammary gland tissue. If they found a mass, they captured a static image and measured the maximum diameter of the mass.

### 2.3. Data Set

In our study, images of normal breast tissue and benign and malignant masses including cystic masses were examined. We also investigated some cases that we had evaluated in our previous study [13].

Ultrasound images in Digital Imaging and Communications in Medicine (DICOM) format were converted to JPEG format using TFS-01 software (Toshiba Medical Systems) and cropped to include the chest wall using Microsoft Paint (Microsoft, Redmond, WA, USA) for analysis. Table 1 shows details of the image characteristics (normal, benign, or malignant), patients’ age, and maximum mass diameter.

For the training phase, we extracted a maximum of 10 different cross-sectional normal bilateral breast images per patient. We collected a total of 531 normal images from 69 patients. Data augmentation (horizontal flip, Gaussian noise injection [μ = 0 and σ = 1], and brightness change [dark; −20% and bright; 20%]) were performed, and 6372 (531 × 12) images were available for training.

For the test phase, we extracted a maximum of four different cross-sectional normal bilateral breast images per patient and only one image of a benign and malignant mass per patient. We used a total of 171 images in 147 patients (51 images of normal tissue in 27 patients, 48 images of benign masses in 48 patients, and 72 images of malignant masses in 72 patients). Table 2 presents the histopathological findings of the masses.

### 2.4. DL Model

DL was performed on a DEEP station (UEI, Tokyo, Japan) containing a graphics processing unit (GeForce GTX 1080; NVIDIA, Santa Clara, CA, USA), central processing unit Core i7-8700 (Intel, Santa Clara, CA, USA), and graphical user interface-based DL tool Deep Analyzer (GHELIA, Tokyo, Japan). Our anomaly detection model was constructed using efficient GAN-based anomaly detection to identify abnormal breast lesions using the training data of normal breast ultrasound images.

Efficient GAN-based anomaly detection is one of the most commonly used anomaly detection methods based on GAN [23]. Conventional GAN is a type of neural network computational model in which two networks are trained simultaneously: one focuses on image generation (= generator) and the other on discrimination (= discriminate).

**Figure 1:**
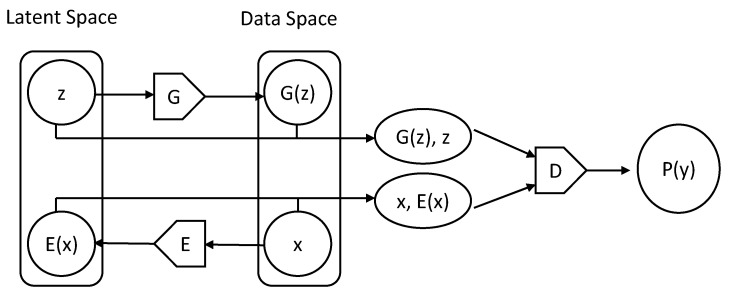
The structure of bidirectional generative adversarial networks.

Our models are based on developed bidirectional GAN methods and simultaneously learn an encoder that maps input samples to a latent space along with a generator and discriminator during training; this enables us to avoid the computationally expensive step of recovering a latent representation during testing [24] (Figure 1). Having trained a model on the normal data to yield a generator, discriminate, and encoder, we then defined an anomaly score that measures how anomalous a test data is based on a convex combination of a reconstruction loss and a discriminator-based loss as follows: Anomaly score = 0.9 × reconstruction loss + 0.1 × discriminator-based loss.

The anomaly colormap was created by deriving the difference between the input test image and the image generated from the trained generator and by converting from grayscale to color scale (jet). When a test image is entered into the trained model, anomaly lesions are visually displayed in red and anomaly score values are displayed on the color map.

The parameters for the generator, discriminator and encoder were the same and are as follows: optimizer algorithm = Adam (clipnorm = 1, clipvalue = 0.5, lr = 0.001, β1 = 0.5, β2 = 0.999, eps = 0.5, decay = o, and amsgrad = False). Breast ultrasound imaging data were set to be input at a pixel size of 128 × 128. The model was trained with 100 epochs.

The figure shows the structure of bidirectional GAN. Generator G transforms a latent representation z into a generated image G(z), and encoder E converts a input sample image x into E(x) and maps it to a latent space. Discriminator D is a binary classifier that calculates the probability that the input sample is real P(y) considering both (G(z), z) and (x, E(x)).

### 2.5. Statistical Analysis

All statistical analysis was performed with the EZR software package version 1.31 (Saitama Medical Center, Jichi Medical University, Saitama, Japan) [25] and the Visualizing Categorical Data package version 1.4-4 with graphical user interface for R software package (version 3.5.1; R Development Core Team, Vienna, Austria).

Using the test dataset, we calculated the sensitivity and specificity of the trained network to distinguish between normal breast tissue, benign masses, and malignant masses. Receiver operating characteristic (ROC) curve was used to calculate the area under the curve (AUC) for performance. An optimal cutoff value was derived that was closest to the upper left corner. The distribution of the anomaly score was analyzed using boxplot. Data are presented as the mean ± standard deviation (SD). Mann–Whitney U-tests were performed to analyze characteristics, including patient age and maximum diameter of mass between benign and malignant masses. One-way analysis of variance and student’s t-test were used to analyze the anomaly scores of the test images. A *p*-value of <0.05 was considered statistically significant.

## 3. Results

Malignant masses were significantly larger than benign masses, and patients with malignant masses were significantly older than those with benign masses (*p* < 0.001; Table 1). The most common histopathology was fibroadenoma in patients with benign masses and invasive ductal carcinoma in those with malignant masses. Thirteen cases were diagnosed as benign by follow-up examination (Table 2).

**Figure 2:**
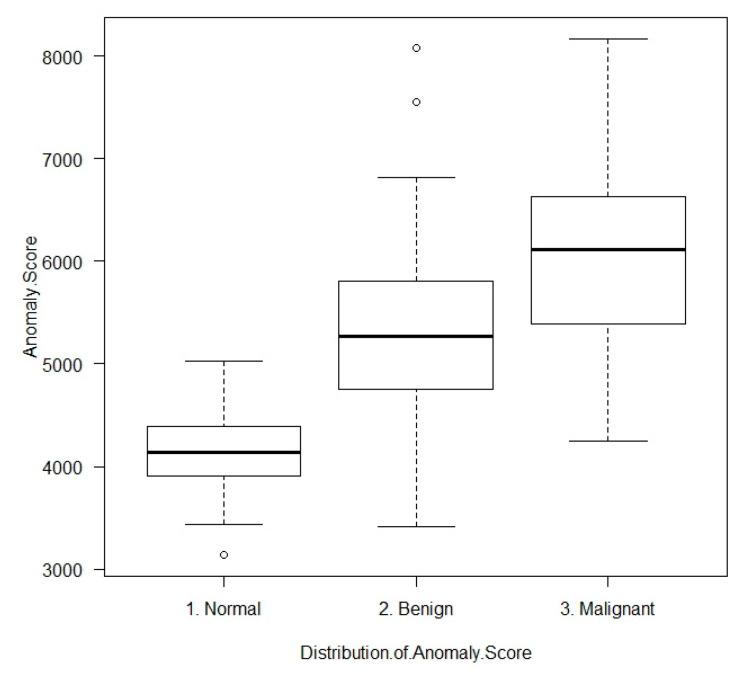
Distribution of anomaly scores.

Table 3 shows the distribution of anomaly scores. The mean ± SD anomaly scores of normal tissues, benign masses, and malignant tissues were 4157.5 ± 418.3, 5283.4 ± 953.3 and 6047.0 ± 842.1. Malignant masses had significantly higher anomaly scores than benign masses (*p* < 0.001), and benign masses had significantly higher scores than normal tissues (*p* < 0.001; Figure 2). Although malignant masses had significantly higher anomaly scores at larger sizes (*p* = 0.025), benign masses showed no significant relationship between size and anomaly scores (*p* = 0.907).

The mean ± SD abnormal score of normal tissue, benign tumor, and malignant tissue were shown by box plot.

**Figure 3:**
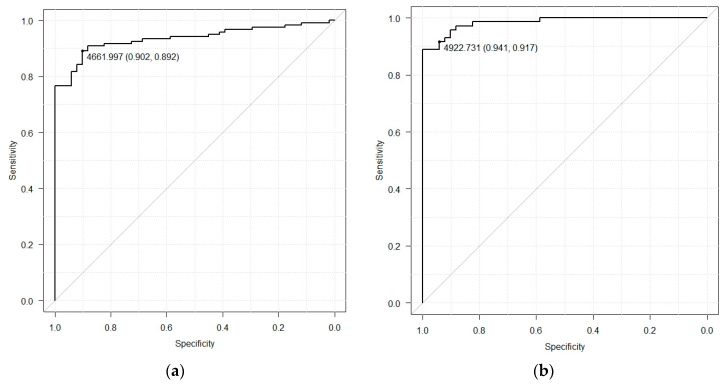

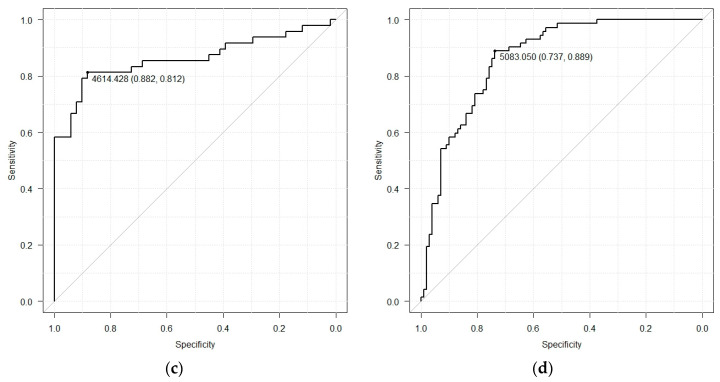
Receiver operating characteristic (ROC) curve. (**a**) ROC curve of normal vs. benign + malignant, (**b**) ROC curve of normal vs. malignant (**c**) ROC curve of normal + benign vs. malignant; and (**d**) ROC curve of benign vs. malignant.

Table 4 lists diagnostic performances with anomaly scores. The diagnostic performance categories of sensitivity, specificity, and AUC were 89.2%, 90.2%, and 0.936 (confidence interval (CI), 0.900–0.972) (cutoff value = 4662) for distinguishing normal tissues from benign and malignant masses (Figure 3a); 91.7%, 94.1%, and 0.985 (CI, 0.969–1.000) (cutoff value = 4923) for distinguishing normal tissues from malignant masses (Figure 3b); 81.2%, 88.2%, and 0.862 (CI, 0.783–0.941) (cutoff value = 4614) for distinguishing normal tissues from benign masses (Figure 3c); and 88.9%, 73.7%, and 0.863 (CI, 0.809–0.917) for distinguishing normal tissues and benign masses from malignant masses (Figure 3d). These data demonstrate that our model displays a high ability to distinguish between normal and abnormal breast tissues, particularly between normal and malignant masses, on ultrasound imaging.

**Figure 4:**
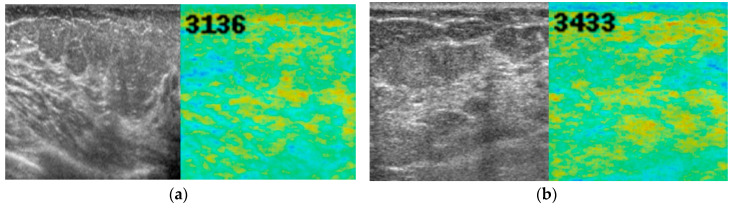
B-mode ultrasound images (left, grey) and anomaly score images (right, color) of normal breast tissue. A 45-year-old woman (**a**) and a 63-year-old woman (**b**) with normal breast tissue. There was almost no abnormal color noted, and the anomaly score was low (3136 and 3433, respectively).

**Figure 5:**
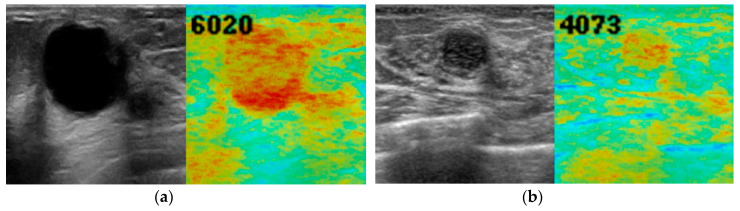
B-mode ultrasound images (left, grey) and anomaly score images (right, color) of breast with benign masses. A 46-year-old woman with intraductal papilloma (**a**) and a 58-year-old woman with fibroadenoma (**b**). There was abnormal red color consistent with the masses. The anomaly score was moderate medium to high (6020 and 4073, respectively).

**Figure 6:**
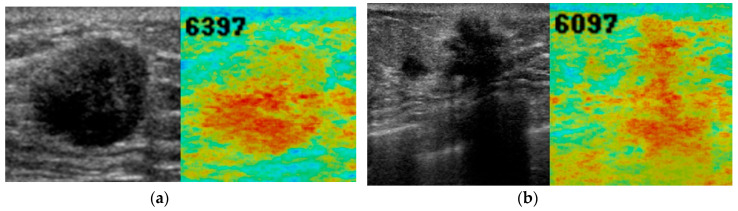
B-mode ultrasound images (left, grey) and anomaly score images (right, color) of breast with malignant masses. A 71-year-old woman with invasive ductal carcinoma (**a**) and a 71-year-old woman with invasive lobular carcinoma (**b**). Abnormal red color was observed on and around the masses. The anomaly score was high (6397 and 6079, respectively).

Figure 4, Figure 5 and Figure 6 show representative images of normal breast tissue (Figure 4a,b), benign masses (Figure 5a,b), and malignant masses (Figure 6a,b).

## 4. Discussion

We demonstrated in previous studies that GAN can generate realistic ultrasound images [11,26]. In the present study, we focused on the detection and differential diagnosis of normal, benign, and malignant breast tissues with ultrasound images using the efficient GAN-based anomaly detection, which applies GAN technology to anomaly detection. and verified its diagnostic accuracy. Our model showed high performance to detect and diagnose anomalous lesions in breast ultrasound images.

For complex, high-dimensional datasets such as images, traditional anomaly detection methods are inadequate. Instead, recent methods based on GAN demonstrate the best anomaly detection performance by leveraging the power of GAN to model high-dimensional data distributions [23]. Efficient GAN is one of the most popular anomaly detection models based on GAN and is constructed on the algorithm that it is considered abnormal when data that deviate from the normal value is put in GAN trained with only normal data [21].

The success of a DL-based method that relies on supervised learning requires large, high-quality, annotated datasets from multiple experts or histopathological diagnoses. Therefore, creating training data is time-consuming and expensive, and it is extremely challenging to collect sufficient amounts of data of rare diseases for training purposes. Furthermore, the performance of these DL models based on supervised learning is highly dependent on the population of the test data. The principal limitation therefore of these models is that they are only useful when testing data similar to the training set.

Anomaly detection is a model that can be established only from normal images that are easy to collect and can be applied to any group. Therefore, it has the potential to overcome these limitations with models that rely on learning with teacher images. In fact, in this study, the DL models could be easily constructed simply by collecting only normal breast ultrasound images.

To our knowledge, although some studies have investigated the usefulness of a GAN-based anomaly detection model for medical images, herein, we report the first clinical application of such a model for breast ultrasound images.

Chen et al. evaluated the detection of anomalous lesions in an unsupervised manner by learning data distribution of healthy subjects’ brain MRI images using two auto-encoder based methods: variational auto-encoder and adversarial auto-encoder models, a type of anomaly detection-based generative network. Lesion images were mapped to lesion-free images by exploring the learned latent space, and then the lesion was highlighted by calculating the pixel-wise absolute intensity difference in the residual image between the two images [27]. Choi et al. used a variational auto-encoder to develop a model trained in an unsupervised manner using a dataset of 353 normal brain positron emission tomography scan images. They showed that the model had a good diagnostic performance to distinguish between normal and abnormal brain images [28].

Our present model using GAN-based anomaly detection showed high diagnostic performance of sensitivity, specificity, and AUC (89.2%, 90.2%, and 0.939, respectively) to distinguish between normal + benign and malignant breast masses on ultrasound. These values are remarkably similar to our past study with a previous model using the convolutional neural networking architecture GoogleNet Inception v2 with supervised training (95.8%, 92.5%, and 0.913, respectively) [13]. However, the present model has some advantages over the previous models. First, the present model was trained on normal images only; thus, we were able to develop it more efficiently with less time and effort than the previous models using images of normal tissues and benign and malignant masses with supervised training. In the previous model, there is a need to adapt to the same population as that in the training data to maintain the diagnostic performance, whereas the present model is applicable to any population.

In the present study, benign masses showed no significant difference between mass size and anomaly score, whereas malignant masses showed a significant difference. This may be because, as the size of the malignant mass increases, infiltration into the surrounding tissues increases and the echo intensity of the surrounding tissues and posterior tissues is greatly affected [29].

This study only examined ultrasound images, and future studies are warranted to examine its correlation with computer-aided design (CAD) systems using other breast imaging modalities such as mammography and magnetic resonance imaging. It would be interesting to correlate the results obtained using ultrasound with those obtained using mammography CAD systems on the same lesions in order to evaluate which of these support systems is more convenient to use [30]. Furthermore, our ultrasound GAN-based anomaly detection can be potentially applied for fusion-imaging systems that use images merged from MRI and ultrasound data [31].

This study has some limitations. First, some masses were diagnosed by follow-up and not by histopathological means. Second, this study was retrospectively conducted at a single institution; therefore, a prospective, multicenter study is needed to verify the results of the present study. Third, the images used in our study were set to be input at a pixel size of 128 × 128. The image processing may have led to a loss of information and may affect the training and diagnostic performance of the models. Fourth, because only three ultrasound systems from two companies were used, further study is needed to verify whether other ultrasound systems perform as well as of the one used in the present study.

In conclusion, our DL model with GAN-based anomaly detection showed high performance to detect and diagnose abnormal lesions on breast ultrasound images. This study suggests that using this DL model for ultrasound can help radiologists detect and diagnose abnormal lesions in breast tissue, thereby putatively reducing the burden on radiologists and increasing the efficiency of diagnostic imaging of breast ultrasound images.

## Figures and Tables

**Table 1:** Characteristics of patients and masses.

Header		Training Data	Test Data		
		Normal	Normal	Benign	Malignant
Patients (*n*)		70	27	48	72
Images (*n*)		531	51	48	72
Age	Mean ± SD (years)	56.8 ± 12.9	52.6 ± 15.8	49.2 ± 12.8	62.3 ± 13.3
	Range (years)	27–85	22–77	25–78	35–92
Maximum Diameter	Mean ± SD (mm)			12.8 ± 7.4	18.2 ± 9.2
	Range (mm)			5–39	5–41

SD, Standard deviation; Comparison was performed with the Mann–Whitney U-test.

**Table 2:** Histopathology of masses.

Test Data	
Benign (*n* = 48)	Malignant (*n* = 72)
Fibroadenoma, 17	Ductal carcinoma in situ, 3
Intraductal papilloma, 8	Invasive ductal carcinoma, 57
Mastopathy, 5	Mucinous carcinoma, 3
Adenosis, 1	Invasive lobular carcinoma, 4
Pseudoangiomatous stromal hyperplasia, 1	Apocrine carcinoma, 2
Radial scar/complex sclerosing lesion, 1	Invasive micropapillary carcinoma, 2
No malignancy, 2	Malignant lymphoma, 1
Unknown, 13 (Diagnosed at follow-up)	

**Table 3:** Distribution of anomaly score.

Header		Mean ± SD	Minimal	Maximum	*p*
Normal		4157.5 ± 418.3	3136	5021	<0.001 ^a^
Benign		5283.4 ± 953.3	3411	8082	
Malignant		6047.0 ± 842.1	4249	8170	
All		5269.1 ± 1107.2	3136	8170	
Benign	<15 mm	5271.8 ± 916.5	3589	7552	=0.907 ^b^
	≥15 mm	5306.7 ± 1054.1	3411	8082	
Malignant	<15 mm	5813.7 ± 763.5	4656	8170	=0.025 ^b^
	≥15 mm	6255.7 ± 863.7	4249	7202	

SD, Standard deviation; Comparison was performed using ^a^ One-way analysis of variance and ^b^ Student’s *t*-test.

**Table 4:** Diagnostic performance with anomaly score.

Header	Sensitivity	Specificity	Cutoff Value (Anomaly Score)	AUC [95% CI]
Normal Vs. Benign + Malignant	89.2%	90.2%	4662	0.936 [0.900–0.972]
Normal Vs. Malignant	91.7%	94.1%	4923	0.985 [0.969–1.000]
Normal Vs. Benign	81.2%	88.2%	4614	0.862 [0.783–0.941]
Normal + Benign Vs. Malignant	88.9%	73.7%	5083	0.863 [0.809–0.917]

AUC, area under the receiver operating characteristic curve; CI, confidence interval.
